# The Role of Dietary Nutrients in Inflammatory Bowel Disease

**DOI:** 10.3389/fimmu.2018.03183

**Published:** 2019-01-15

**Authors:** Kohei Sugihara, Tina L. Morhardt, Nobuhiko Kamada

**Affiliations:** ^1^Division of Gastroenterology, Department of Internal Medicine, University of Michigan, Ann Arbor, MI, United States; ^2^Division of Pediatric Gastroenterology, Hepatology and Nutrition, Department of Internal Medicine, University of Michigan, Ann Arbor, MI, United States

**Keywords:** inflammatory bowel disease (IBD), diet, microbiota, immunity, intestinal barrier

## Abstract

Inflammatory bowel disease (IBD) is a chronic and relapsing inflammatory disease of the gastrointestinal tract. Although the precise etiology of IBD remains incompletely understood, accumulating evidence suggests that various environmental factors, including dietary nutrients, contribute to its pathogenesis. Dietary nutrients are known to have an impact on host physiology and diseases. The interactions between dietary nutrients and intestinal immunity are complex. Dietary nutrients directly regulate the immuno-modulatory function of gut-resident immune cells. Likewise, dietary nutrients shape the composition of the gut microbiota. Therefore, a well-balanced diet is crucial for good health. In contrast, the relationships among dietary nutrients, host immunity and/or the gut microbiota may be perturbed in the context of IBD. Genetic predispositions and gut dysbiosis may affect the utilization of dietary nutrients. Moreover, the metabolism of nutrients in host cells and the gut microbiota may be altered by intestinal inflammation, thereby increasing or decreasing the demand for certain nutrients necessary for the maintenance of immune and microbial homeostasis. Herein, we review the current knowledge of the role dietary nutrients play in the development and the treatment of IBD, focusing on the interplay among dietary nutrients, the gut microbiota and host immune cells. We also discuss alterations in the nutritional metabolism of the gut microbiota and host cells in IBD that can influence the outcome of nutritional intervention. A better understanding of the diet-host-microbiota interactions may lead to new therapeutic approaches for the treatment of IBD.

## Introduction

Inflammatory bowel disease (IBD) is a chronic and relapsing inflammatory condition of the gastrointestinal tract. Crohn's disease (CD) and ulcerative colitis (UC) are the two principal types of IBD. The prevalence of IBD has been increasing worldwide with the highest incidence found in Western countries (1). Although the precise etiology of IBD remains unclear, interactions between genetic and environmental factors are associated with its pathogenesis (2, 3). Advances in next-generation gene sequencing technology have led to the identification of over 160 genetic polymorphisms associated with the risk for IBD (4). Since the number of IBD patients in developing countries has rapidly increased in the past several decades, in concert with industrialization and westernization of lifestyle, it is unlikely that susceptibility genes are the primary driver of rising rates of IBD (5). Therefore, it is likely that environmental exposure is the most significant risk factor in IBD.

Among environmental factors, accumulating evidence suggests that dietary nutrients contribute to the pathogenesis of IBD (6, 7). Specifically, diets rich in fat and protein, common in the western world and countries with similar lifestyles, have been identified as risk factors for the development of IBD. Hence, nutritional intervention, which aims to reduce the intake of potential nutritional hazards, is a treatment option in IBD that induces and extends disease remission (8). Moreover, some dietary nutrients can potentiate the host immune system and intestinal barrier function, which in turn protect the host from disease. Thus, providing beneficial nutrients, while limiting nutritional hazards, is a key strategy for successful dietary therapies designed for the treatment of IBD. In addition to affecting host immunity and intestinal barrier function, dietary nutrients have an impact on the composition and function of the gut microbiota. The altered gut microbiota can, in turn, influence host physiology and disease. Furthermore, the metabolism of host immune and non-immune cells, as well as that of the gut microbiota, are known to change during inflammation. Hence, the demand for certain nutrients by the host and/or the microbiota may be changed in IBD (9–11). A more thorough understating of the complex interplay among dietary nutrients, host immunity, and the gut microbiota is required to increase the effectiveness of dietary interventions used to treat IBD. Herein, we review the current knowledge of the role of dietary nutrients in the development and the treatment of IBD.

## Dietary Amino Acids and IBD

Dietary amino acids act as key regulatory factors in cellular and microbial metabolic pathways. They also play important roles in gut homeostasis. Intestinal inflammation affects several metabolic pathways and disturbances in amino acid metabolism are observed in IBD patients. Amino acid metabolic profiles in the blood, urine, feces, and intestinal tissues are also altered in IBD patients and correlate with the severity of disease (Table 1). Additionally, metagenomic studies have revealed that amino acid biosynthesis genes are downregulated and amino acid transporter genes are upregulated in the gut microbiome of IBD patients, indicating that the gut microbiota lessens the production of amino acids and increases the rate of their utilization (11, 25). In addition to bacteria, host immune cells also utilize amino acids differently during inflammation. For instance, certain amino acids are critical for T cell effector function as well as the proliferation of macrophages (26, 27). Thus, the demand for certain amino acids by host cells and the gut microbiota may increase as a result of inflammation.

**Table 1:** Comparison of amino acid levels between healthy subjects and IBD patients.

**Sample**	**Patients**	**Increase**	**Decrease**	**References**
Serum	HC: 60, UC: 60		UC: Asn, Asp, Gln, His, Trp, Val, Ile, Thr, Pro, Ser, Met, Glu, Tyr, Lys,	(10)
Serum	HC: 20, UC: 20, CD: 20		CD: Leu, Lys, Val, Arg, Ser, Gln	(12)
Serum	HC: 40, UC: 20, CD: 20	UC: Lys, Ile	UC: Tyr, Val, Ser	(13)
		CD: Pro, Arg, Gly, Ile	CD: Val	
Serum	HC: 17, UC: 24, CD: 19	IBDa: Leu, Ile, Gly, Phe,	IBDa: His	(14)
Serum	HC: 17, UC: 22, CD: 21	UC: Asp, Gly	UC: Asn, Gln, Glu, His, Trp	(15)
		CD: Ala, Asp, Gly, Met, Pro	CD: Gln, His, Trp	
Serum	HC: 29, UC: 25, CD: 26	UC: Arg, Ile, Ser, Pro	UC: Trp	(9)
		CD: Arg, Ile, Ser, Pro	CD: Trp	
Plasma	HC: 102, UC: 102, CD: 102	UC: Pro	UC: Val, Leu, Met, His, Trp, Phe, Asn, Tyr	(16)
			CD: Val, Leu, Ile, Thr, Lys, Met, His, Phe, Asn, Gln, Gly, Tyr	
Urine	HC: 21, IBD: 21	IBD: Gly, Asp, Cys, Glu, Ile, Met, Pro, Val		(17)
Urine	HC: 60, UC: 30, CD: 30		IBD: His, Lys, Asp	(18)
Urine	HC: 40, UC: 20, CD: 20	UC: Trp, Thr, Arg	UC: Ser, Phe	(13)
		CD: Thr		
Urine	HC: 17, UC: 24, CD: 19	IBDa: Gly	IBDa: Ala	(14)
Feces	HC: 29, UC: 25, CD: 26	UC: Asp, Gly, Trp, Ser, Thr, Asn, His, Phe, Ala	CD: Asp, Thr, Asn, His	(9)
		CD: Gly, Trp, Ser, Phe, Ala		
Feces	UC: 15, CD: 15, HV: 15	UC: Ala, Gly, His, Ile, Leu, Lys, Phe, Pro, Ser, Thr, Trp, Tyr, Val		(19)
		CD: Ala, His, Leu, Phe, Trp, Tyr, Val		
Feces	HC: 13, UC: 10, CD: 10	UC: Gln, Lys		(20)
		CD: Ala, Ile, Leu, Lys, Val		
Feces	HC: 21, UC: 41, CD: 44	UCa: Ile, Leu, Val, Lys, Ala		(21)
		CDa: Ile, Leu, Val, Lys, Ala, Tyr, Phe, Gly		
Feces	HC: 51, UC: 82, CD: 50	UC: Gly, Phe	UC: Glu	(22)
		CD: Ala, Phe		
Colonic biopsies	HC: 17, UC: 22, CD: 21		Inflamed: Ala, Asp, Glu, Gln, Gly, Ile, Leu, Lys, Met, Phe, Pro, Ser, Thr, Tyr, Val	(15)
Colonic biopsies	HC: 26, UC: 31, CD: 26	UCa: Arg/Leu/Lys	UCa: Ile/Leu/Val, Ala, Glu/Gln	(23)
			CDa: Ile/Leu/Val, Ala, Glu/Gln	
Colonic biopsies	HC: 25, UC: 68	UCa: Glu, Gln, Asp		(24)

*HC, healthy control; IBD, inflammatory bowel disease; UC, ulcerative colitis; CD, Crohn's disease; UCa, active ulcerative colitis; CDa, active Crohn's disease*.

### Tryptophan

**Figure 1:**
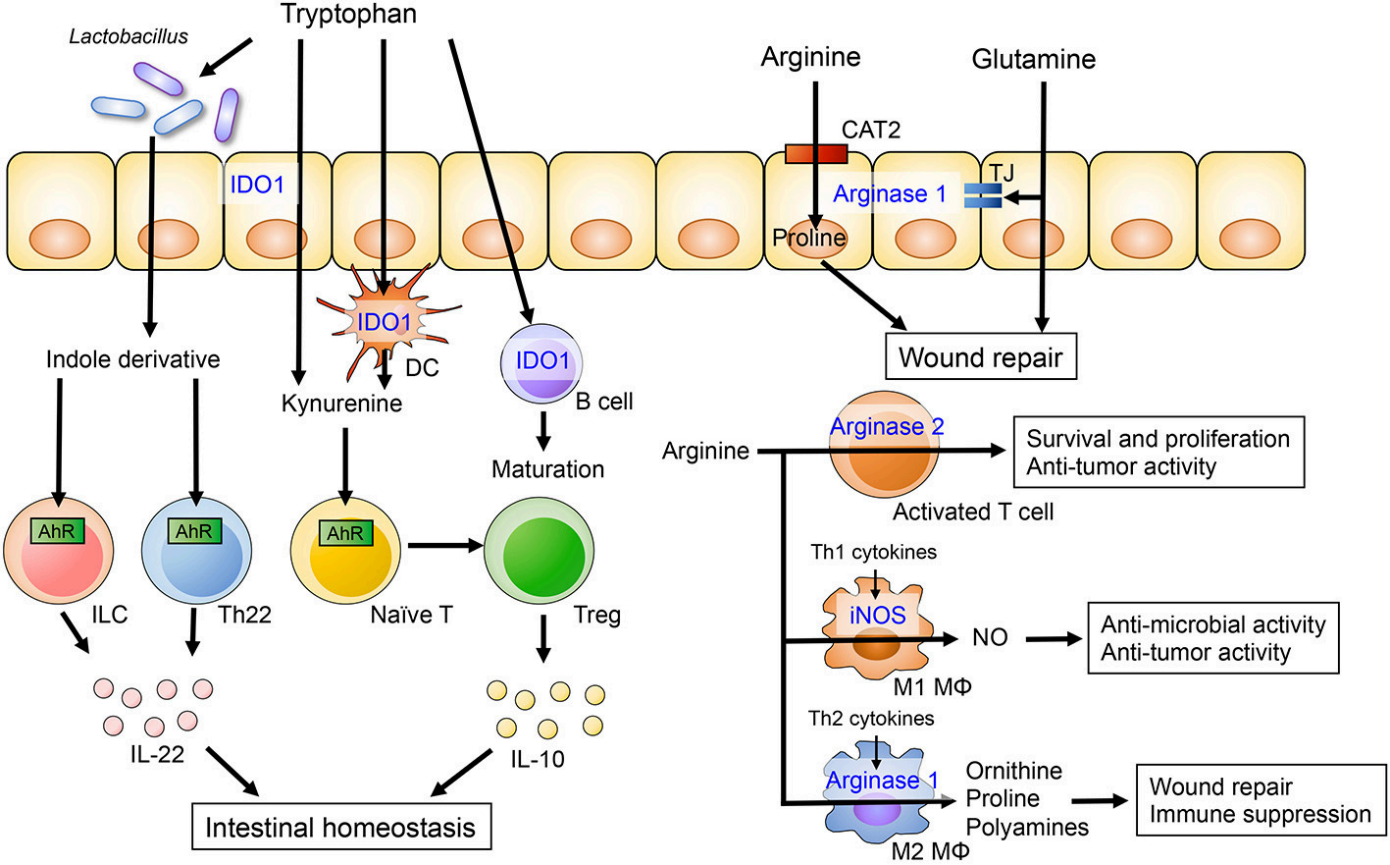
The role of dietary amino acids in intestinal homeostasis. Dietary tryptophan is metabolized to kynurenine or indole derivatives by host cells or the gut microbiota, respectively. Kynurenine promotes the differentiation of Treg and induces IL-10 production by Treg cells through AhR. *Lactobacillus* species are capable of catabolizing tryptophan into indole derivatives that are ligands for the AhR. Activation of AhR in gut-resident T cells and ILC enhances production of IL-22, which in turn potentiates mucosal barrier integrity. Arginine and glutamine metabolism in intestinal epithelial cells is associated with epithelial barrier and intestinal wound repair. Arginine also plays an important role of the immune system. Arginine is catabolized by iNOS in M1 macrophages and by arginase II in M2 macrophages. The arginine metabolisms regulate anti-microbial and -tumor activity and immune suppression in M1 and M2 macrophages, respectively.

Tryptophan is an essential amino acid and a common constituent of protein-based foods, such as fish, meat, and cheese. It is utilized in the synthesis of nicotinamide derivatives, indole derivatives and serotonin (28). Tryptophan metabolites, such as kynurenine, indole-3-aldehyde, and indole-3-acetic acid, can act as ligands for the aryl hydrocarbon receptor (AhR), a critical regulator of immunity and inflammation involved in adaptive immunity and intestinal barrier function (Figure 1) (29, 30). In a murine model of colitis, mice fed a tryptophan-deficient diet showed exacerbation of colitis accompanied by increased weight loss and reduced levels of antimicrobial peptides (31). Conversely, dietary supplementation with tryptophan and tryptophan metabolites ameliorated intestinal inflammation in experimental colitis (31–35).

Indoleamine 2,3 dioxygenase-1 (IDO1) is ubiquitously expressed in epithelial cells, dendritic cells and macrophages. IDO1 is the first step in the kynurenine pathway, a major route for tryptophan catabolism (28). IDO1 regulates the differentiation and maturation of adaptive immune cells (36). Kynurenine is an initial metabolite of IDO1-mediated tryptophan catabolism and the kynurenine/tryptophan ratio is a surrogate marker of IDO1 activity. A recent clinical study has shown that serum tryptophan levels are lower and the kynurenine/tryptophan ratio is elevated in IBD patients compared to healthy controls (37). Additionally, IDO1 mRNA expression in colonic tissues is significantly higher in IBD and correlates with disease severity, suggesting the kynurenine pathway is upregulated in IBD. The expression of IDO1 is also higher in murine models of experimental colitis and has been shown to regulate inflammatory response (38). Since kynurenine is known to have anti-inflammatory properties, elevated levels of IDO1 in IBD may be a counter reaction to inflammation. Indeed, in the 2,4,6-trinitrobenzene sulfonic acid (TNBS)-induced colitis model, disease severity is exacerbated in IDO1 deficient mice or mice receiving an IDO inhibitor (38, 39). IDO1 also attenuates intestinal inflammation in other models of colitis, including graft vs. host disease (40) and the *Citrobacter rodentium* infection model (41). Kynurenine promotes the differentiation of regulatory T cell (Treg) and induces IL-10 production by Treg cells through AhR activation (30, 42). Likewise, kynurenine-mediated AhR activation increases expression of the IL-10 receptor in intestinal epithelial cells (35). IL-10 signaling regulates mucosal wound repair through WNT1-inducible signaling pathway protein 1, suggesting that kynurenine plays a role in mucosal wound repair (43). Additionally, kynurenine supplementation ameliorates body weight loss, intestinal permeability and histology in the model of dextran sodium sulfate (DSS)-induced colitis (35).

Peripheral immune activation can affect the systemic metabolism of tryptophan and emotional behavior. Activation of T cells in *Pdcd1*^−/−^ mice, which lack the inhibitory programmed cell death protein 1 (PD-1), has been shown to affect the blood metabolic profile (44). Specifically, it results in an increase of amino acid uptake and intracellular accumulation of free amino acids, resulting in the reduction of amino acid levels (especially tryptophan) in the blood. Tryptophan is essential for the synthesis of the neurotransmitter serotonin, which regulates many aspects of animal behavior, including anxiety, aggression and fear (45). Brain serotonin levels are lower in *Pdcd1*^−/−^ mice. Accordingly, these animals are prone to anxiety and exhibit enhanced fear responses. Collectively, this evidence suggests that activation of T cells causes a systemic metabolic shift, resulting in abnormal emotional behavior. Importantly, dietary supplementation of tryptophan ameliorates these behavioral abnormalities. Anxiety and depression are more common in patients with IBD and symptoms stemming from these conditions tend to be more severe during periods of active disease (46). Thus, tryptophan is an important nutrient that plays a role in the regulation of inflammation and maintenance of mental health.

In addition to tryptophan metabolism in the host, tryptophan metabolites generated by the gut microbiota also contribute to the regulation of mucosal homeostasis (47). A genome-wide association study has found that caspase recruitment domain-containing protein 9 (CARD9), an adaptor protein involved in apoptosis and antifungal immunity, is a susceptibility gene for IBD (4). CARD9 deficient mice produce reduced amounts of IL-22, a cytokine with an important role in maintaining mucosal immunity and integrity (48), and are more susceptible to colitis (49). Interestingly, the gut microbiota of CARD9 deficient mice lack certain bacteria, such as *Lactobacillus reuteri* and *Allobaculum*, that are capable of catabolizing tryptophan into indole derivatives (49). Hence, dysbiotic microbiotas in CARD9 deficient mice do not generate indole derivatives, which promote mucosal IL-22 production through AhR activation (49). Consistently, reduced levels of AhR ligands are also typically observed in the microbiotas found in IBD patients, particularly in those with the CARD9 risk alleles associated with IBD (49). Thus, gut dysbiosis in the context of IBD may lead to compromised tryptophan catabolism, which in turn influences IL-22-mediated mucosal protection.

### Arginine

Arginine is a semi-essential amino acid and a substrate for 4 enzymes including arginases, nitric oxide synthases (NOS), arginine-glycine amidinotransferase and arginine decarboxylase (50). Arginases have two isoforms, arginase I and arginase II, both of which metabolize L-arginine to NO and citrulline. Arginase I is highly expressed in the liver, whereas arginase II is expressed in the brain, kidneys, mammary glands and intestine. nitric oxide synthases (NOS) catalyzes the synthesis of NO from arginine. There are three NOS isoenzymes: neuronal nitric oxide synthase (nNOS), inducible nitric oxide synthase (iNOS), and endothelial nitric oxide synthase (eNOS). Arginine uptake into cells is mainly mediated by the cationic amino acid transporter (CAT) family of proteins encoded by the solute carrier (SLC).

Alterations in arginine metabolism have been reported in animal models of colitis as well as IBD patients, and arginine supplementation has been shown to ameliorate experimental colitis (51–53). A prospective cohort study has demonstrated that colonic arginine levels are decreased in UC patients and they correlate with disease severity (51). Altered levels of arginine in the colonic tissue are associated with increased mRNA expression of the arginine metabolic enzymes arginase II and iNOS. Although previous animal and clinical studies have revealed contradictory findings regarding arginine transporter expression during inflammation, arginine transport is critical for the maintenance of gut homeostasis (51–53). Wounding of intestinal epithelial cells has been shown to alter the expression of genes related to arginine transport and metabolism *in vitro* (52). CAT2, unlike CAT1, mediates intestinal wound repair and cell migration (54). Therefore, a CAT2 deficiency renders mice susceptible to DSS-induced colitis (53). Arginine catabolism also promotes wound repair. L-arginine metabolites, including proline and ornithine, are associated with restitution of intestinal epithelial integrity. Hence, supplementation of arginine during the recovery phase of DSS-induced colitis alleviates intestinal inflammation and promotes the migration of colonic epithelial cells (52). Thus, arginine metabolism plays a crucial role in the resolution of inflammation and, therefore, arginine supplementation could be used to promote mucosal healing in IBD.

Arginine has also been suggested to be crucial for immune cell activation and function. Arginase II is up-regulated in activated T cell, resulting in enhanced CD4^+^ and CD8^+^ T cell survival and anti-tumor activity (55). Depletion of extracellular arginine has been found to impair aerobic glycolysis, T cell proliferation and cytokine production (56–58). Additionally, the deletion of argininosuccinate 1 (ASS1), an enzyme utilized in the *de novo* synthesis of arginine, blunts Th1 and Th17 cell polarization even in the presence of extracellular arginine (59). Similarly, arginine metabolism may play a role in the regulation of macrophage functions. It has been reported that macrophages can undergo polarization toward the M1 or M2 phenotype based on environmental polarization cues. Stimulation with LPS plus IFN-γ induces M1 polarization, whereas Th2 cytokines, such as IL-4, IL-10, and IL-13, induce M2 polarization (60, 61). Interestingly, these two types of macrophages catabolize arginine differently. M1 macrophages express iNOS, which converts arginine to NO and citrulline. Both of these arginine metabolites are involved in the elimination of intracellular pathogens and tumor cells (62). In contrast, M2 macrophages express arginase I, which regulates the synthesis of proline and polyamines from ornithine, and is critical for wound healing and immune suppression (63). While a number of studies have demonstrated the importance of arginine metabolism in immune cells, the role of arginine in intestinal immunity is poorly understood. Thus, more research is necessary to understand how arginine metabolism shapes intestinal immune response during inflammation.

### Glutamine

Glutamine is considered a conditionally essential amino acid because its consumption is increased during conditions of catabolic stress, including trauma, sepsis and post-surgery recovery (64). Glutamine is the most important fuel for enterocytes and immune cells, and has beneficial effects on clinical outcomes (65). Activation of lymphocytes results in a metabolic reprogramming that switches energy metabolism from oxidative phosphorylation to aerobic glycolysis and glutaminolysis, the process by which cells convert glutamine into TCA cycle metabolites (66, 67). Thus, glutamine metabolism and the demand for glutamine during inflammation may be increased in certain cells. Indeed, glutamine levels in the colonic tissue of IBD patients in remission are decreased compared to control subjects. This difference is even more considerable during active disease (15, 68). In addition to the colonic tissue, blood glutamine levels are also diminished in IBD patients and experimental animal models of colitis (10, 69). Oral or rectal supplementation of glutamine attenuates intestinal inflammation, intestinal fibrosis, and colitis-associated colon tumorigenesis in various models of colitis (69–73). Glutamine treatment significantly improves histology results, reduces oxidative stress and cytokine production via downregulation of the NF-κB and STAT signaling pathways (72). Glutamine also regulates epithelial integrity. Glutamine has been implicated in the preservation of gut barrier function, maintenance of epithelial tight junction integrity (74) and modulating paracellular permeability (75). In contrast, glutamine deprivation or inhibition of glutamine synthase significantly increase paracellular permeability and decrease the expression of tight junction proteins (76, 77). Glutamine is also associated with the proliferation of Lgr5-positive intestinal stem cell and subsequent crypt expansion. In intestinal organoid culture, the deprivation of glutamine suppresses epithelial proliferation (78). Replenishment of culture medium with supplementation of glutamine rescues the proliferation of epithelial cells via the activation of mammalian target of rapamycin (mTOR) (78). However, glutamine deprivation does not affect the proportions of Paneth and goblet cells, indicating that certain amino acids may support the differentiation and proliferation of intestinal epithelial cells in a lineage-specific manner. Although glutamine supplementation has been shown to be beneficial in murine models of experimental colitis, the utility of this amino acid in IBD patients remains poorly understood. Clinical studies showed that glutamine-supplemented enteral nutrition and total parenteral nutrition did not improve therapeutic outcomes for pediatric CD, adult CD, and adult UC patients (79, 80).

### Other Amino Acids

Metabolome analyses have shown that the levels of other amino acids, such as histidine, glycine, and threonine, are altered in the serum and intestinal tissues of IBD patients (10, 13, 15). These amino acids have also been reported to be beneficial to IBD patients and in models of experimental colitis. Histidine is a natural amino acid and is one of conditionally essential amino acids. Histidine acts as a scavenger of hydroxyl radicals and singlet oxygen (81), and hence can suppress oxidative stress in intestinal epithelial cells (82). Moreover, dietary histidine ameliorates intestinal inflammation in the IL-10-deficient cell transfer model of colitis through the inhibition of NF-κB activation, following the down-regulation of pro-inflammatory cytokine production in macrophages (83). In IBD patients, the levels of histidine in the blood and intestinal tissues are markedly decreased compared to control subjects (10, 15). A recent clinical study has shown that the plasma histidine level is a predictor of relapse risk in UC patients (84). Glycine has been reported to be protective against various kinds of organ injuries as well as intestinal inflammation. Dietary glycine improves histology and dampens the expression of pro-inflammatory cytokines in TNBS- and DSS-induced colitis (85). Threonine is an essential amino acid with roles in maintaining intestinal homeostasis. It is important for mucosal barrier function, which prevents luminal bacteria from penetrating the mucosal tissue (86). Defects in intestinal mucosal barrier function are observed in IBD patients and experimental colitis, enabling gut bacteria to penetrate the intestinal mucosa (87, 88). Mucins contain high levels of threonine, serine and proline (89). Consequently, mucin synthesis requires robust amounts of these amino acids and restriction of dietary threonine impairs intestinal mucin synthesis (89). In contrast, dietary supplementation of threonine, serine, proline and cysteine ameliorates intestinal damage in DSS-treated rats, presumably by improving mucosal barrier function (90).

Intestinal inflammation changes the metabolic requirements of host cells and the gut microbiota. The up-regulation of amino acid metabolic pathways is one of the major inflammation-related metabolic shifts in the host and the microbiota (11, 44, 55). In other words, host cells and resident microbes have an increased demand for certain amino acids in the context of IBD. Thus, dietary supplementation of these sought-after amino acids is a key strategy for the treatment of IBD. However, it is noteworthy to mention that other amino acids may fuel pro-inflammatory responses in IBD and restriction of certain amino acids can attenuate intestinal inflammation. Amino acid deficiency is sensed by a serine/threonine-protein kinase called general control non-derepressible 2 (GCN2) (91). GCN2 suppresses pathogenic Th17 responses by inhibiting ROS-mediated activation of the inflammasome (92). Therefore, when GCN2 is deleted in a tissue-specific manner from intestinal epithelial cells and CD11c^+^ antigen-presenting cells, the animals experienced more severe weight loss and intestinal inflammation, indicating that both intestinal epithelial cells and antigen-presenting cells mediate the protective effects of GCN2 during colitis. Notably, amino acid starvation, induced by the consumption of a low-protein diet or a leucine-deficient diet, can suppress DSS-induced colitis through the activation of GCN2 (92). Since deprivation of other amino acids, such as tryptophan, tends to enhance intestinal inflammation (31), it is likely that each amino acid plays a distinct role in the promotion and/or suppression of intestinal inflammation. Thus, a more thorough understanding of the specific roles different amino acids play in IBD and supplying the optimal amount of anti-inflammatory amino acids while limiting the consumption of pro-inflammatory amino acids can lead to the development of effective amino acid-based dietary interventions.

## Carbohydrate

Carbohydrates are an important source of nutrients for both host cells and the gut bacteria. However, host cells largely lack the capacity to digest complex poly-saccharides (93). Rather, dietary fibers and other carbohydrate polymers are degraded and fermented by the gut microbiota into mono-saccharides as well as various by-products. Recent accumulating evidence indicates these gut microbial byproducts can modulate the host immune system and intestinal barrier function, thus playing a crucial role in intestinal homeostasis (94). Therefore, the intake of indigestive carbohydrates (dietary fibers) benefits the host. On the other hand, gut dysbiosis may compromise the metabolic activities of the gut microbiota, resulting in impaired generation of the protective microbial byproducts. Furthermore, high intake of undigested and fermentable carbohydrates, such as mono- and di-saccharides, induces various gastrointestinal symptoms. In this section, we summarize the roles of dietary fiber and fermentable carbohydrates in intestinal homeostasis and the management of IBD symptoms.

### Fibers

**Figure 2:**
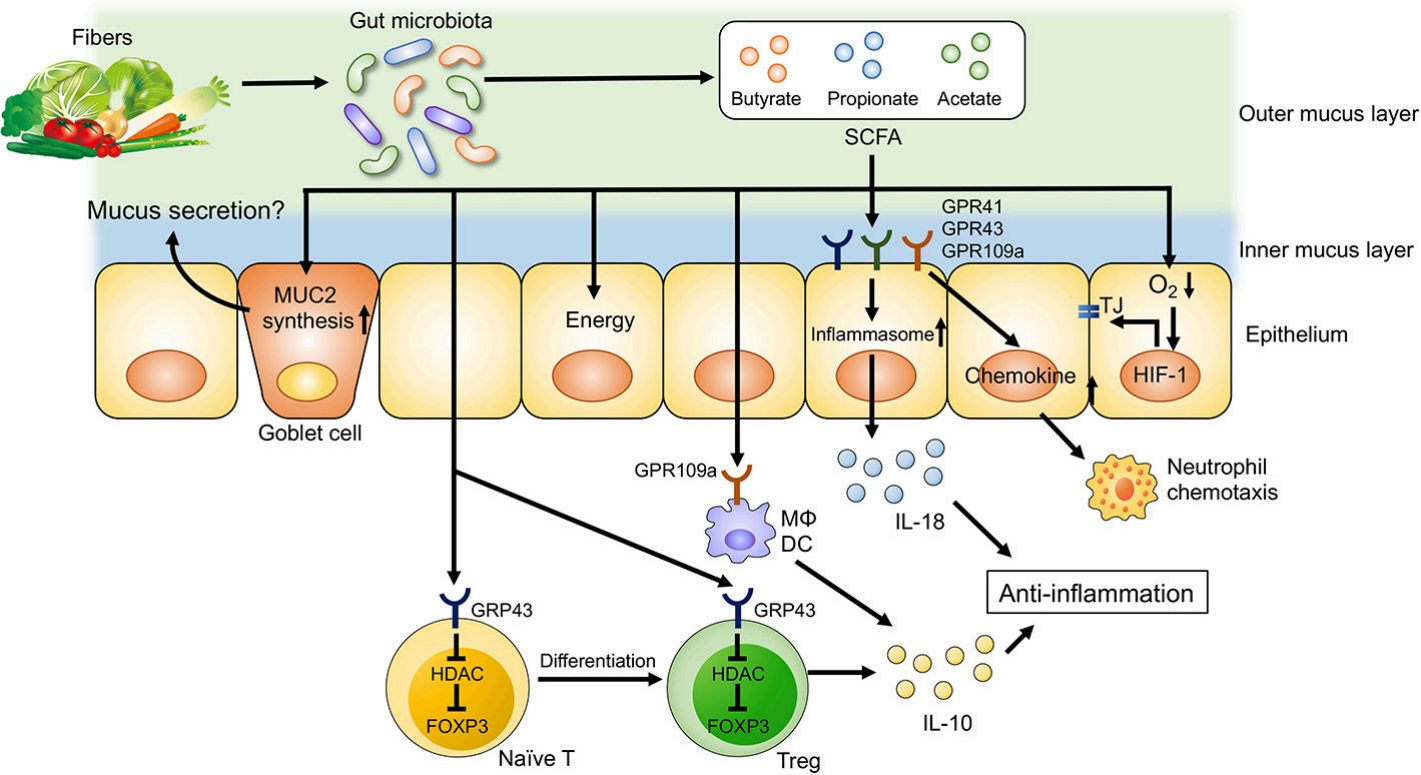
Dietary fiber-derived SCFAs regulate intestinal homeostasis. Dietary fiber-derived SCFAs serve as energy substrates for colonocytes. Likewise, SCFAs regulate intestinal barrier function and immune system through GPCRs signaling. SCFAs promote the differentiation into Treg cells and the production of IL-10 from Treg cells through GPR43. SCFA facilitate inflammasome activation in colonic epithelial cells through GPR43, resulting in an IL-18 production that is critical for anti-inflammation and epithelial repair. SCFAs also regulate intestinal barrier function via enhancing the expression of tight junction proteins and the synthesis of MUC2.

Dietary fibers are complex carbohydrates consisting of both soluble and insoluble components. Dietary fibers are not digested or absorbed by host cells, as mammalian cells largely lack the enzymes necessary to degrade them. Instead, dietary fibers are subjected to bacterial fermentation in the gastrointestinal tract. Although a wide range of bacteria ferment dietary fibers, each bacterium has a substrate preference based on its enzymatic activity. For example, the human gut symbionts *Bacteroides thetaiotaomicron* and *B. ovatus* can degrade a wide variety of glycans, whereas some *Bacteroides* species are restricted to one or only a few types (95). Thus, dietary intervention can remodel the gut microbial composition by customizing the content of dietary fibers. Also, bacterial byproducts generated through the fermentation of dietary fibers have various effects on host immune cells and intestinal barrier function. Short-chain fatty acids (SCFAs), such as acetate, propionate and butyrate, are the major end products of microbial fermentation of dietary fibers (Figure 2) (93). Dietary fiber-derived SCFAs are key energy substrates utilized by colonocytes (96). Additionally, SCFAs act as signaling molecules via G-protein-coupled receptors (GPRs), such as GPR41, GPR43, and GPR109a. SCFAs can regulate the differentiation of immune cells and immune responses through GPRs. SCFAs have been shown to promote anti-inflammatory responses through the activation of GPRs, as mice deficient in GPR43 and GPR109a develop more severe DSS-induced colitis (97, 98). Furthermore, GPR109a signaling promotes anti-inflammatory properties in colonic macrophages and dendritic cells as well as inducing the differentiation of regulatory type T cells, such as Treg cells and IL-10-producing T cells (97). Since SCFAs are generated by the gut microbiota through fermentation of dietary fibers, germ-free (GF) mice display lower levels of colonic Foxp3^+^ Treg cells (99). Butyrate is known to regulate histone acetylation, which in turn up-regulates the expression of Foxp3. Transcriptional factor Foxp3 is a lineage specification factor of Tregs that has been shown to prevent the development of colitis in a T cell transfer model of colitis (100). Collectively, SCFAs are important immunomodulatory molecules that exert numerous beneficial effects on host metabolism and immunity.

In addition to their regulatory function, SCFAs and, by extension, dietary fibers are also important for mucosal barrier function. Butyrate enhances intestinal epithelial barrier function via hypoxia inducible factor-1 (HIF-1) that regulates the integrity of epithelial tight junctions (101, 102). Likewise, butyrate treatment in mice facilitates the assembly of tight junction proteins and increases the synthesis of MUC2 protein, the main component of intestinal mucus (103, 104). Thus, a lack of dietary fibers may compromise epithelial integrity and mucus production due to insufficient SCFA generation, resulting in impaired intestinal barrier function (100, 101). In contrast, butyrate is known to suppress the proliferation of intestinal stem cells. Butyrate inhibits histone acetylation and enhances the promoter activity for the negative cell-cycle regulator Foxo3 in intestinal stem cells (105). Increased expression of Foxo3 in intestinal stem cells results in delayed wound repair after mucosal injuries (105). Notably, a recent report has unveiled that consumption of a low fiber diet leads to the disruption of intestinal barrier function through a mechanism that is independent of SCFAs. In the absence of dietary fibers, some commensal bacteria, such as *Akkermansia muciniphila*, utilize host mucus glycans to meet their energy needs (106). As a result, these mucolytic bacteria become the predominant species within the gut microbiota (106). Importantly, a bloom of mucolytic bacteria results in the degradation of the colonic mucus layer that renders the host susceptible to enteric pathogens, such as *C. rodentium* (106).

Gut dysbiosis observed in IBD patients is primarily characterized by reduced bacterial diversity, the enrichment of the phylum of *Proteobacteria* and a lower abundance of *Firmicutes* and *Bacteroidetes* phyla (107). IBD-associated gut dysbiosis is accompanied by functional changes in the gut microbiota that affect its ability to ferment dietary fibers. The abundance of butyrate-producing bacteria, such as *Roseburia hominis* and *Faecalibacterium prausnitzii*, is decreased in IBD patients (11, 108, 109) and, therefore, fecal SCFAs levels are lower in IBD patients compared to controls (20). On the other hand, recent clinical trials did not demonstrate overt therapeutic benefits associated with butyrate enemas in UC patients (110, 111). These results suggest that IBD patients might have functional impairments involving butyrate utilization in addition to its impaired generation, likely due to gut dysbiosis. For instance, several studies have reported that butyrate oxidation and the expression of genes involved in butyrate oxidation are diminished in the intestinal mucosa of IBD patients (112–115), indicating that butyrate cannot be used properly in the inflamed gut. Moreover, butyrate uptake by colonocytes is impaired in IBD patients. Although butyrate uptake is mediated by monocarboxylate transporter 1 (MCT1), the transcription of *Mct1* is downregulated upon stimulation with IFN-γ and TNF-α, thereby reducing the uptake of butyrate (116). Consistently, expression levels of *Mct1* mRNA negatively correlate with the degree of intestinal inflammation in IBD (116).

Although numerous animal studies have reported that dietary fiber supplementation attenuates intestinal inflammation (117–119), only limited evidence is available from clinical trials involving IBD patients (120). Prebiotic fibers might promote the growth of protective members of the gut microbiota. However, there is no study that provides statistically significant evidence for the efficacy of prebiotic fibers as treatment for IBD (121). It implies that fiber treatment is not effective for active IBD. Regarding this notion, it has been reported that intestinal inflammation down-regulates carbohydrate metabolism in the gut microbiota (11, 25). Thus, it is possible that gut microbes are not able to utilize supplied fibers efficiently in the inflamed gut. Consistently, consumption of a high fiber diet does not attenuate colitis, while pretreatment with the same diet can prevent the development of colitis (119). Thus, metabolic activities of the microbiota, which might be altered as a result of disease, may determine the usefulness of administrating prebiotic fibers to IBD patients. In addition to disease status, the initial composition of the microbiota may influence the efficacy of dietary fiber treatment. For example, dietary fiber can improve postprandial glucose metabolism in healthy individuals, but they are divided into responders and non-responders (122). The abundance of *Prevotella*, a bacterial genus capable of digesting fibers, is higher in responders compared to non-responders. In other words, a high fiber diet is not beneficial in individuals who do not have sufficient numbers of fiber-degrading bacteria in their gut microbiota. Taken together, personalized nutritional management will likely be a key component of any successful therapeutic approach for IBD since intestinal inflammation results in both compositional and functional changes of the gut microbiota.

### Elimination of Fermentable Carbohydrate

Several dietary therapies that eliminate specific carbohydrates have been developed and evaluated for induction of remission, maintenance and improvement of functional symptoms in IBD. Symptoms of IBD commonly include abdominal pain, discomfort, rectal bleeding, and a change in stool consistency and frequency during active periods. However, similar gastrointestinal symptoms can also be observed during periods of remission, even in the absence of clinical inflammation (123). These functional-like gastrointestinal symptoms are negatively correlated with the quality of life (QOL) experienced by IBD patients (124). Naturally, a more effective control of functional-like gastrointestinal symptoms is needed to improve the QOL of IBD patients. Dietary therapies for these functional-like gastrointestinal symptoms have recently been developed. These therapies include a low Fermentable Oligo-saccharides, Di-saccharides, Mono-saccharides And Polyols (FODMAP) diet and a Specific Carbohydrate Diet (SCD) (8). We will review the effects of a low-FODMAP diet on IBD symptoms, because there is emerging clinical evidence suggesting the usefulness of this approach.

A diet low in FODMAP is used to manage symptoms in patients with irritable bowel syndrome (IBS) (125). A randomized, double-blind, placebo-controlled, cross-over study has revealed that the administration of fructans, unlike galacto-oligosaccharides or sorbitol, exacerbates functional-like gastrointestinal symptoms in quiescent IBD (126). A recent meta-analysis has shown that a low FODMAP diet can reduce gastrointestinal symptoms in patients with quiescent IBD (127). Although the precise mechanism by which FODMAPs promotes functional-like gastrointestinal symptoms is unclear, FODMAP intake is associated with increased luminal water content and colonic gas production (125). Whereas, most carbohydrates are completely digested and absorbed in the small intestine, some carbohydrates are not. Unabsorbed carbohydrates, such as fructose, polyols, and lactose, alter intestinal osmolality and increase water content in the small intestine. Other unabsorbed carbohydrates, including fructans and galacto-oligosaccharides, are fermented by the bacteria in the colon, leading to gas production. High levels of luminal water and gas result in luminal distension, thereby causing functional-like gastrointestinal symptoms. In an animal study, a high FODMAP diet increased intestinal permeability and altered the composition of microbial communities, leading to increased luminal levels of LPS (128). Inhibition of Toll-like receptor (TLR) activation by LPS ameliorates intestinal barrier function and visceral nociception. Moreover, since fecal LPS is higher in IBS patients compared to healthy subjects, a low-FODMAP diet improves IBS symptoms by reducing the levels of fecal LPS. Dietary intervention is an exciting topic in the current IBD research. Further studies are needed to unravel the mechanism of action and the long-term effects of a low FODMAP diet.

## Fatty Acids

The Western diet is characterized by high intake of fats. There is evidence that links increased fat intake to the pathogenesis of IBD. Dietary fat comprises saturated fatty acids (SFAs), monounsaturated fatty acids (MUFAs), and polyunsaturated fatty acids (PUFAs). Most fatty acids, except for linoleic acid and alpha-linolenic acid which are essential fatty acids, can synthesize in the body. Fatty acids are important energy sources and membrane constituents, and their biological activities influence both the immune system and the gut microbiota. A number of studies have revealed that high-fat diets change the composition of the gut microbiota and impair intestinal barrier function, resulting in intestinal and systemic inflammation (129–131). Furthermore, consumption of a diet containing high levels of fat and sugar promotes the colonization of adherent-invasive *E. coli* (AIEC), an IBD-associated pathobiont (132). A recent systematic literature review of epidemiological data has shown that high dietary intakes of total fats, SFAs, omega-6 (n-6) and PUFAs are associated with an increased risk of both CD and UC (133). Similar to amino acids and carbohydrates, abnormal fatty acid profiles and metabolism are observed in the blood and intestinal mucosa of IBD patients (134). On the other hand, dietary supplementation with specific fatty acids can improve perturbed fatty acids profiles and intestinal inflammation (135). We summarize the roles of fatty acids in the pathogenesis and treatment of IBD.

### Saturated Fatty Acids (SFAs)

SFAs, mainly myristic acid, palmitic acid, and stearic acid, are found in animal fat-containing products, such as meat, butter, whole milk, and other dairy products. Although a connection between saturated fats and the risk of IBD has been uncovered by small case–control studies, prospective cohorts have not identified a statistical association, suggesting the relationship is more complex. In animal studies, a diet high in SFAs induces colonic inflammation, including increased expression of pro-inflammatory cytokines and enhanced intestinal permeability in a microbiota- or TLR4-dependent manner (129–131). Similarly, a diet high in SFAs exacerbates intestinal inflammation in experimental models of colitis (130, 136). A milk-derived diet high in saturated fats, unlike a diet high in polyunsaturated fats, promotes the expansion of a sulphite-reducing pathobiont, *Bilophila wadsworthia*, and aggravates Th1-mediated colitis in IL-10 deficient mice (136). Milk-derived fat promotes taurine conjugation of hepatic bile acids, thereby leading to the accumulation of sulfur that aids the growth of *B. wadsworthia*.

In a murine model of DSS-induced colitis, the level of a saturated fat 1-stearoyl-sn-glycero-3-phosphorylcholine (stearoyl-LPC) in the blood is increased compared to control mice, whereas the unsaturated fat 1-oleoyl-sn-glycero-3-phosphorylcholine (oleoyl-LPC) is decreased following DSS treatment. Thus, intestinal inflammation shifts the balance between saturated LPCs and monounsaturated LPCs (135). The perturbation of the lipid serum profile is caused by decreased expression of stearoyl-CoA desaturase 1 (SCD1) in the liver. SCD1 is an enzyme found in the endoplasmic reticulum. It is responsible for the biosynthesis of oleic acid (18:1) and palmitoleic acid (16:1) from stearic acid (18:0), and palmitic acid (16:0), respectively (137). Saturated LPCs, unlike their monounsaturated counterparts, increase IL-1β production in human monocytes (138). Consistently, SCD1-deficient mice are more susceptible to DSS and exacerbated colitis can be ameliorated by means of oleic acid supplementation (135).

### Monounsaturated Fatty Acids (MUFAs)

MUFAs are classified as fatty acids containing a single double bond. Oleic acid, the most common omega-9 MUFA, is found in various vegetable oils, such as olive oil and canola oil. The levels of MUFAs, including oleic acid, are significantly reduced in IBD patients compared to control subjects, both systemically (blood) and locally (intestinal mucosa) (139, 140). Epidemiologically, the Mediterranean diet containing high levels of MUFAs, especially oleic acid, is well-recognized for its beneficial effects on metabolic syndrome and cardiovascular health (141). The effects of MUFAs on IBD remain unresolved. There are conflicting reports regarding the therapeutic potential of MUFAs in IBD. A large prospective cohort study has demonstrated that dietary oleic acid intake is inversely associated with UC development (142). In contrast, other studies have shown that a high intake of MUFAs increases the risk for the development of UC and CD. Several animal studies have consistently demonstrated that supplementation of oleic acid or olive oil attenuates intestinal inflammation in DSS-induced colitis (135, 143).

### Polyunsaturated Fatty Acids (PUFAs)

PUFAs are classified into omega-3 (n-3) and n-6 families. These two families are thought to have different effects on inflammation. Conventionally, n-6 PUFAs are considered to be pro-inflammatory. Linoleic acid, the major PUFA in the diet, is converted to arachidonic acid, a precursor of inflammatory mediators such as prostaglandins and leukotrienes (134). N-6 PUFAs are incorporated into phospholipids found in cellular membranes. Multiple members of the phospholipase family of enzymes, such as phospholipase A2 (PLA2), hydrolyze n-6 PUFAs during inflammation. Arachidonic acid is a primary target for PLA2 and is converted to inflammatory mediators involved in pro-inflammatory cell signaling events. The levels of PLA2 are elevated in the mucosal biopsies of CD and UC patients (144), resulting in increased hydrolysis of arachidonic acid and the subsequent generation of inflammatory mediators. In contrast, n-3 PUFAs appear to be critical for the suppression of inflammation. α-linolenic acid, an essential n-3 PUFA, is a precursor of eicosapentaenoic acid (EPA) and docosahexaenoic acid (DHA), both of which are contained in fish oils. N-3 PUFAs compete with n-6 PUFAs in the substrate pool of the lipoxygenase pathway, inhibiting the production of inflammatory mediators downstream of n-6 PUFAs (134). Likewise, pro-resolving mediators are generated from n-3 fatty acids via several enzymatic reactions (145). Resolvin E1 (RvE1) is an anti-inflammatory lipid mediator derived from EPA through aspirin-acetylated cyclooxygenase-2 (COX2) and microbial cytochrome P450 (146, 147). RvE1 inhibits leukocyte infiltration and IL-12 production by dendritic cells and macrophages (148, 149). Consistent with the anti-inflammatory effects on immune cells, the administration of RvE1 attenuates TNBS- and DSS-induced colitis in mice (146, 149). Moreover, n-3 PUFAs can directly inhibit the TLR4 and NF-κB signaling pathways, leading to the down-regulation of pro-inflammatory genes, such as COX2 and TNF-α (150, 151). Thus, an appropriate balance of n-6/n-3 PUFAs is important for the regulation of inflammation.

A Western diet typically contains high levels of n-6 PUFAs and low levels of n-3 PUFAs (152). The skewed ratio between the intake of n-6 PUFAs and n-3 PUFAs is considered a risk factor for IBD (133). Although previous studies have examined the effect of n-3 fatty acid supplementation in prevention and treatment of IBD, the outcomes were inconsistent in both human IBD and animal models of colitis. In the animal studies, supplementation with n-3 fatty acids found in fish oil attenuated intestinal inflammation (153, 154), whereas another study showed that n-3 fatty acids exacerbated colitis (155). This disparity may have been caused by various confounding factors, such as control diet, administration period and sensitivity to oxidation. Another report demonstrated that fat-1 transgenic mice are protected against DSS-induced colitis (156). Since fat-1 transgenic mice have increased endogenous levels of n-3 PUFAs, the protective phenotype observed in this mouse strain may be due to n-3 PUFAs (156). In human IBD, a systematic review does not support the notion that supplementation of n-3 fatty acids can induce and maintain remission of IBD (157). The latest Cochrane review has reached a similar conclusion (158). Interestingly, several studies have demonstrated that different genotypes may be associated with the variable response to nutritional intervention with n-3 PUFAs. For example, genetic polymorphisms of TNF-α and PPARα have been associated with an altered response to nutritional intervention with n-3 PUFAs (159, 160). In IBD, genetic polymorphisms, such as CYP4F3 and Caspase 9+93C/T, modify the association between dietary fatty acid intake and risk of IBD (161, 162). Since fatty acid utilization may vary depending on the genetic background, personalized nutritional management is likely required to maximize the efficacy of n-3 PUFAs supplementation in IBD.

## Phytochemicals

Phytochemicals are a wide range of natural compounds found in plants. Accumulating evidence suggest that phytochemicals have beneficial effect on several chronic diseases (163). A number of polyphenols, including flavonoids, phenolic acids, stilbenes, and lignans, have been shown to influence the gut microbiota and intestinal immunity. For example, curcumin, a hydrophobic polyphenol derived from *Curcuma longa*, displays a wide variety of biological functions, such as anti-inflammatory, anti-cancer, and anti-oxidant activities (164). Curcumin suppresses cytokine production by macrophages and intestinal epithelial cells via the inhibition of NF-κB activation (165, 166), and thus administration of curcumin ameliorates DSS- and TNBS-induced colitis (167, 168). Polyphenols including curcumin are not efficiently absorbed from the small intestine, and therefore polyphenol complexes contained in diet have limited bioavailability (169). Given this evidence, a large portion of unabsorbed polyphenols are likely transported to the large intestine where they interact with the colonic gut microbiota. Of note, the gut resident bacteria are capable of catabolizing polyphenols and degrading them into small fragments (170). For instance, curcumin is converted into tetrahydrocurcumin by curcumin-converting enzyme purified from *E. coli* (171). Curcumin-derived tetrahydrocurcumin can prevent colitis and colitis-associated colon cancer in mice (167, 172). Recent epidemiological study reported a potential association of polyphenol in CD (173). Moreover, clinical trials show that curcumin supplementation is effective for the induction and maintenance of remission in UC patients (174, 175).

## Food Additives

Recent widespread use of processed food increases consumption of food additives. Emulsifiers are complex molecules that are incorporated into most processed foods to enhance texture and stability. It has been implied that the increased consumption of emulsifier is associated with IBD pathogenesis (176). In recent studies, the consumption of dietary emulsifier disrupted host-microbiota interaction and promoted intestinal inflammation and colon carcinogenesis (177, 178). Although precise mechanism remains incompletely understood, dietary emulsifiers foster the blooms of mucolytic bacteria, such as *Ruminococcus gnavus* and *Akkermansia muciniphila* (177). Importantly, the negative impact of dietary emulsifiers is not observed in GF mice, suggesting that the compositional and functional modulation of the gut microbiota by emulsifiers play a key role in their adverse effect (177). Other food additives, for example artificial sweeteners, also induce gut dysbiosis (179, 180). The consumption of artificial sweeteners promotes the expansion of *Proteobacteria* and increases the infiltration of bacteria into the ileal lamina propria in CD-like ileitis model mice (180). Moreover, dietary phosphate, the main component of many food additives, enhances intestinal inflammation through the activation of NF-κB in macrophages (181). These studies suggest that many food additives may be associated with the risk of IBD. In fact, epidemiological and clinical studies reported that processed meats and beverages are risk factors for the onset and relapse of IBD (182, 183). However, it is difficult to ascertain whether food additives are risk factors for developing IBD, because most food frequency questionnaires cannot fully evaluate the consumption of food additives.

## Conclusion and Future Direction

The relationship between dietary nutrients and intestinal homeostasis is complex. It is influenced by a myriad of interactions between host immunity, the intestinal epithelium and the gut microbiota. Moreover, intestinal inflammation changes cellular and microbial metabolisms, adding another layer of complexity to an already complex system (Table 2). The demand for certain nutrients necessary for the maintenance of intestinal homeostasis is likely altered in IBD. The scarcity or overabundance of some nutrients can disturb intestinal homeostasis even further, thus exacerbating the disease. Genetic polymorphisms also influence the efficacy of nutritional intervention. Thus, an in-depth knowledge of a patient's genetic predispositions, gut microbial compositions, disease activity and type (i.e., ileal, colonic, etc.) will be required to maximize the effectiveness of nutritional intervention approaches in IBD.

**Table 2:** Changes of dietary nutrient availavility in the host and gut microbiota of IBD.

**Host or gut microbiota**	**Nutrient availability**	**Mediator**	**IBD**	**Related nutrient**	**References**
Host	Metabolic enzyme	IDO1	UCa, CDa > UCia, CDia, Control	Tryptophan	(37)
		Arginase II, iNOS	UCa > UCia, Control	Arginine	(51)
		ACSM3, ACADS, ECHS1	UC, CD > Control	SCFA	(113, 115)
	Transporter	MCT1 (SLC16A1)	UC, CD < Control	SCFA	(116)
		B0AT1 (SLC6A19)	UCa, CDa < UCia, CDia, Control	Tryptophan, Neutral amino acid	(37)
		CAT2 (SLC7A2)	UCa < Control	Arginine, Cationic amino acids	(51)
	SNP	CARD9	UC, CD	Tryprophan	(49)
		CYP4F3	UC	Fatty acids	(162)
		Caspase 9	CD	Fatty acids	(161)
		PPAR-γ	CD	Fatty acids	(161)
		Fas ligand	CD	Fatty acids	(161)
Gut microbiota	Microbial composition	Butyrate producung bacteria	UC, CDa < Control	Fiber, SCFA	(25, 108, 109)
	Metabolic pathway	Carbohydrate metabolism	CDi > Control	Carbohydrates	(11)
			UC Inflamed < UC normal	Carbohydrates	(25)
		Amino acid metabolism	UC Inflamed > UC normal	Amino acids	(25)
		Lysine/Arginine biosynthesis	UC, CD < Control	Amino acids	(11)
		Lipid metabolism	CDi < Control	Fatty acids	(11)
			UC Inflamed > UC normal	Fatty acids	(25)
		Butyrate and propionate metabolism	CDi < Control	SCFA	(11)
	Transport system	Carbohydrate transport	CDi > Control	Amino acids	(11)
		Lysine/arginine/ornithine transport	UC, CD > Control	Amino acids	(11)

*UCa, active ulcerative colitis; UCia, inactive ulcerative colitis; CDa, active Crohn's disease; CDia, inactive Crohn's disease; CDi, CD with ileal; UC inflamed, tissues of inflamed lesion from UC; UC tissues of normal lesion from UC; Normal DO1, Indoleamine 2,3 dioxygenase-1; iNOS, inducible nitric oxide synthase; ACSM3, acyl-CoA synthetase medium chain family member 3; ACADS, Acyl-CoA dehydrogenase; ECHS1, enoyl-CoA hydratase, short chain 1; MCT1, monocarboxylic acid transporter 1; B0AT1, systemB(0) neutral amino acid transporter 1; CAT2, cationic amino acid transporter 2; CARD9, caspase recruitment domain 9; PPAR-γ, Peroxisome Proliferator-Activated Receptor gamma*.

## Author Contributions

All authors listed have made a substantial, direct and intellectual contribution to the work, and approved it for publication.

### Conflict of Interest Statement

The authors declare that the research was conducted in the absence of any commercial or financial relationships that could be construed as a potential conflict of interest.
